# Anonymous-authentication scheme based on fog computing for VANET

**DOI:** 10.1371/journal.pone.0228319

**Published:** 2020-02-13

**Authors:** Mu Han, Shuai Liu, Shidian Ma, Ailan Wan

**Affiliations:** 1 School of Computer Science and Communication Engineering, Jiangsu University, Zhenjiang, China; 2 Automotive Engineering Research Institute Jiangsu University, Zhenjiang, China; Wuhan University, CHINA

## Abstract

Privacy protection in vehicular ad hoc networks (VANETs) has always been a research hotspot, especially the issue of vehicle authentication, which is critical to ensure the safe communication of vehicles. However, using the real identity in the process of authentication can easily result in a leak of the privacy information of the vehicles. Therefore, most existing privacy-protection schemes use anonymous authentication and require one-to-one communication between vehicles and the trusted authority (TA). However, when the number of vehicles is too large, network congestion can take place. In addition, the process of updating the anonymous by the TA or the vehicle itself, can result in both poor real-time performance and leakage of the system master key. To solve these problems, this study proposes a fog-computing-based anonymous-authentication scheme for VANETs; the scheme reduces the communication burden of the TA by enabling self-authentication between vehicles and road-side units (RSUs), thus improving the vehicle-authentication efficiency. For updating the anonymous, we design a fog-computing-based pseudonym-updating and -tracking strategy, which guarantees real-time communication and reduces the instances of re-authentication interactions for legitimate vehicles. The experimental results show that the scheme not only meets the privacy-protection requirements of VANETs but also offers better performance than that of the existing anonymous-authentication schemes.

## Introduction

The vehicular ad hoc network (VANET) is a core component of the intelligent transportation system and plays an indispensable role in many aspects such as improving communication efficiency and reducing traffic accidents [1]. The nodes of VANET comprise the following two parts: the onboard unit (OBU), which is installed in vehicles and the road-side unit (RSU), which is located on the road-side [2]. Using the OBU, vehicles can achieve the vehicle-to-vehicle, vehicle-to-infrastructure, and broadcast communications [3] for comfortable and safe services (e.g., weather information, entertainment-related internet service, and traffic accidents) [4]. However, owing to the characteristics of the open network environment and dynamic network topology, the VANET faces many challenges in the field of secure communication. As the precondition of secure communication, the authentication of vehicles guarantees the legitimacy of each communication node for vehicles to achieve secure communication. Therefore, the authentication of vehicles is particularly important in the VANET. However, there are still some challenges: 1) how to implement an efficient and secure authentication scheme between the vehicles and RSU [5]; 2) how to protect the privacy of users during the process of authentication. Therefore, designing an efficient and secure anonymous-authentication scheme has wide applications [6–7].

In recent years, researchers have proposed many authentication schemes for VANET in order to address this problem. Most of these schemes achieved security authentication based on anonymous. Meanwhile, to avoid tracking attacks, vehicles need to change their pseudonyms frequently. At the beginning, these existing schemes can verify the identities of vehicles in the VANET, by which malicious vehicles could be prevented from communicating with other legitimate vehicles or RSUs, and, thus, the privacy information of the vehicles could also be protected. However, it is difficult to accomplish efficient authentication when the number of authentication requests increases in a short time, and if the certificate revocation list (CRL) is large. Subsequently, the transmission delay gets longer when the size of the CRL becomes larger [8]. During this period, malicious vehicles can continually compromise the VANET. Also, broadcasting the CRL to other vehicles will disclose the privacy information of the revocation vehicles, as the legal vehicles have all the pseudonyms of the revoked vehicles. Considering the issues of inefficient authentication and costs caused by the CRL, many related scholars proposed several efficient authentication schemes using the hash message authentication code (HMAC), which prevents the attackers from changing the content of the messages sent by legitimate vehicles or RSUs [9]. Moreover, if an anonymous vehicle in the VANET system becomes malicious, its privacy should be revoked by the trusted authority (TA) and revealed to other vehicles [10], so that it can no longer be anonymous; this is done to protect the performance of the system. Thus, the revocation scheme has been considered as very essential to retain other users as honest in the VANET [11].

In this study, we proposed a novel authentication scheme that leverages fog-computing architecture to protect the privacy of vehicles (i.e., achieving anonymity) for the VANET. The following are the main contributions of this study:

1. A two-way anonymous-authentication scheme, which is based on anonymity, is designed, in which the RSU and the vehicle do not need the TA in order to participate in the process of identity authentication, thereby reducing the burden of the trusted center, as well as the authentication delay.
2. By introducing fog computing to generate and update the anonymity of vehicles, legitimate vehicles do not need to authenticate all the RSUs in the driving period, thereby reducing the times of authentications between legitimate vehicles and RSUs.

The rest of this paper is structured as follows: Section 2 details the related work; Section 3 provides the system model; Section 4 presents the proposed scheme; Section 5 provides the security analysis of this paper. Section 6 analyses the performance of the proposed protocol. Finally, Section 7 concludes this paper.

## Related work

The existing authentication schemes for the VANET are mainly based on pseudonyms in order to achieve efficient and secure anonymous authentication.

Lu et al. [12] proposed a pseudonym-based effective conditional privacy-protection protocol, which is based on bilinear mapping, to obtain the conditional privacy of vehicles. However, the RSU has high latency while generating pseudonyms. In addition, the RSU is usually vulnerable to physical attacks and hazards, thereby not guaranteeing security very well. Huang et al. [13] proposed an efficient pseudonymous authentication-based conditional privacy protocol for VANETs (PACP), in which the TA first generates a long-term pseudonym for vehicles, following which the vehicles obtain a "token" from the RSU. Finally, the vehicles generates its own pseudonym to achieve anonymous communication. However, the limitation of PACP is that during token generation, the RSU does not know any information regarding vehicles, and it is the only entity to generate tokens in the VANET; therefore, the complete reliability of tokens cannot be guaranteed. Furthermore, Skim et al. [14] proposed a pseudonym-based conditional privacy-protection authentication protocol, which improves the efficiency of node-identity authentication by reducing the time-consuming mapping operation. However, the frequent authentication process increases not only the computation cost and authentication delay but also the burden for the authentication agency. In addition to privacy protection, how to achieve effective authentication of vehicles is also an important challenge for the contemporary VANET. Therefore, researchers proposed pseudonym-based batch authentication schemes, such as the revocable group batch authentication scheme (RGB) [15], the anonymous batch authentication and key agreement [16], and the authentication scheme for VANETs with batch verification (BVV) [17] under the random oracle model.

In addition, for designing anonymous VANET authentication scheme based on pseudonym, some papers choose group signature to achieve anonymous authentication of the node identity. Among them, Lin et al. [18] introduced group signature into the VANET for the first time, thereby preventing the leakage of the user's privacy information in the process of identity authentication. However, in the entire process, frequent group key updates increase the computational overhead; therefore, the scheme cannot meet the high efficiency requirements of the VANET. Furthermore, Zhong et al. [19] proposed an efficient group signature scheme with revocation (GSR), which combines the subset cover framework with Camenisch–Stadler. However, the group signature scheme also faces some open security problems; i.e., group administrators are not protected, and the selection of relevant vehicle group administrators may endanger the privacy of all the group members.

However, the pseudonym-based authentication scheme does not face the security threat caused by the group signature scheme, and the former is more efficient than the latter [20]. However, in the pseudonym-authentication-based VANET, one-to-one communication is required between vehicles and the TA. In addition, when the number of vehicles is too large, network congestion is caused easily. Besides, the process of anonymous update by the TA or by the vehicle itself can easily cause both poor real-time performance and leakage of the system master key.

In this study, we provide a fog-computing-based anonymous-authentication scheme for the VANET; the scheme reduces the communication burden of the TA by performing self-authentication between vehicle and RSUs, thereby improving the efficiency of vehicle authentication. For an anonymous update, we design a fog-computing-based pseudonym-updating and tracking strategy, which guarantees real-time communication and reduces the instances of re-authentication interactions for legitimate vehicles.

## System overview

### System model

**Fig 1:**
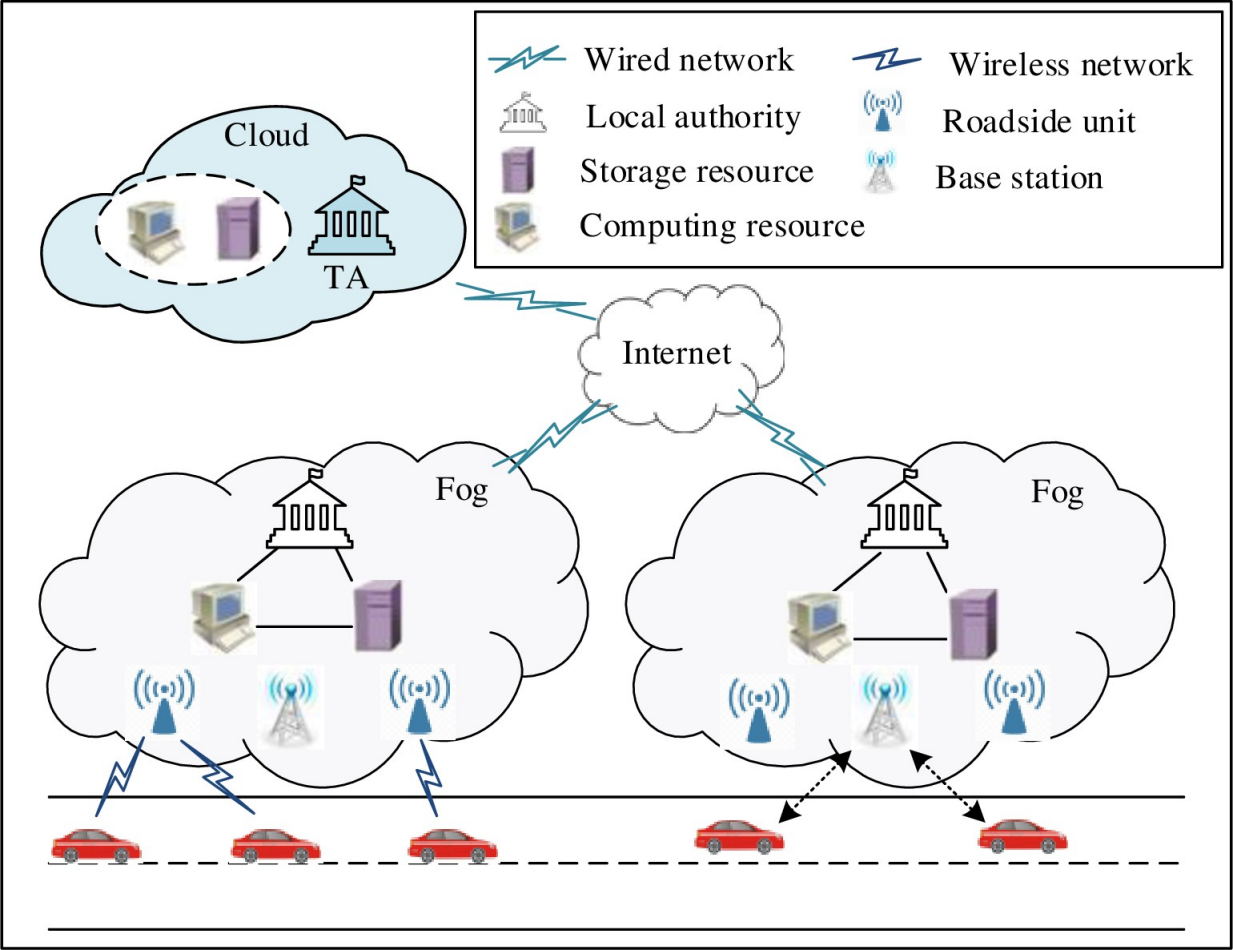
Network model.

The system model of this study is depicted in Fig 1, which consists of three major layers, namely, the cloud layer, the fog layer, and vehicles.

1. Cloud layer: It is the trust authority of the entire system and has the powerful ability to calculate and store a large amount of information. Clouds mainly include the TA, computing resources, and storage resources. In this study, first, the TA is responsible for registering and managing the local authorities (Las) and vehicles, as well as allocating certification and system parameters to them simultaneously. Second, it also exposes the true identities (TIDs) of the vehicles in a traffic dispute.
2. Fog layer: In cloud computing, the elements of the network infrastructure (such as RSU and base station) are deployed near the edge of the network, and they are interconnected to form a fog layer. In the network infrastructure, there is a dedicated local fog server to connect to the Internet wirelessly, and to provide a wireless interface for vehicles to access computing and storage resources. These fog servers use the network-function virtualization technology in order to virtualize the physical resources in the fog infrastructure, to build virtual machines for computing instances. In addition, to realize flexible resource allocation among fog servers, virtual machines are dynamically created, migrated, loaded, and destroyed according to different network states, by using the network technology defined by software [21]. On the basis of these technologies, the fog layer is implemented in the real scene. In this study, each fog mainly consists of five parts, namely, the LA, RSU, base station, computing resources, and storage resources. The LA is responsible for generating and updating the anonymous information of vehicles and, subsequently, recording it in the storage resources of the corresponding fog layer, thereby distributing the anonymous information to the corresponding vehicles through RSU, and, thereafter, uploading the generated anonymous information to the cloud layer. The RSU is a fixed roadside communication unit, which communicates with the LAs and vehicle through both wired and wireless networks. This study assumes that the RSU is completely trusted and is used to verify the validity of the vehicle identity, and that the anonymity generated by the LAs is forwarded to the vehicle.
3. Vehicles: Each vehicle is equipped with an OBU, which shares some value information (for example, traffic safety warnings) with RSUs and other legitimate vehicles, through wireless communication technology. Each OBU possesses a tamper-proof device for storing public keys, private keys, and other sensitive and confidential information. In addition, a global positioning system (GPS) provides the location information of vehicles.

### Attack model

Owing to the openness of network environment in the VANET, it is inevitable to face the following attacks:

1. Impersonation attack: Attackers may pretend to be a legal vehicle or RSU in order to cheat other legal nodes.
2. Message repudiation attack: When the authorities reveal the real identity of the attacker, the attacker can repudiate the malicious information sent previously.
3. Error message attack: Attackers send some error messages to affect the judgment of users, which, in turn, may lead to accidents.
4. Privacy attack: Attackers obtain sensitive information of vehicles by analyzing the content of messages.
5. Message replay attack: Attackers replay valid messages that had been sent previously, to disturb transportation.

For the above-mentioned attacks, the authentication feature can resist the impersonation attack; the traceability feature can resist the message non-repudiation attack; the integrity and unforgeability features can resist the error message attack and the message replay attack; and the anonymity feature can resist the privacy attack.

### Definitions and assumptions

#### Discrete Logarithm (DL) Problem

Let *P* be the generator of $G_{1}$, for $a\in z_{p}^{*}$. Given *P* and *aP*, compute *a*.

The probability of $D_{DL}$ success is defined as follows:

$${Adv}_{DL}=Pr[D_{DL}(P,aP)=a]$$

DL Assumption: ${Adv}_{A}^{DL}$ is a negligible value for all the PPT algorithm $D_{DL}$.

Computational Diffie–Hellman (CDH) problem

Let *P* be the generator of $G_{1}$, for all $a,b\in z_{p}^{*}$. Given (*P*, *aP*, *bP*), compute *abP* by using the probabilistic polynomial time algorithm A.

The probability of A success is defined as follows:

$${Adv}_{A}^{CDH}=\mathrm{Pr}[A(P,aP,bP)=abP:a,b\in z_{p}^{*}]$$

CDH Assumption: ${Adv}_{A}^{CDH}$ is a negligible value for all the PPT algorithm A.

## Proposed system

**Fig 2:**
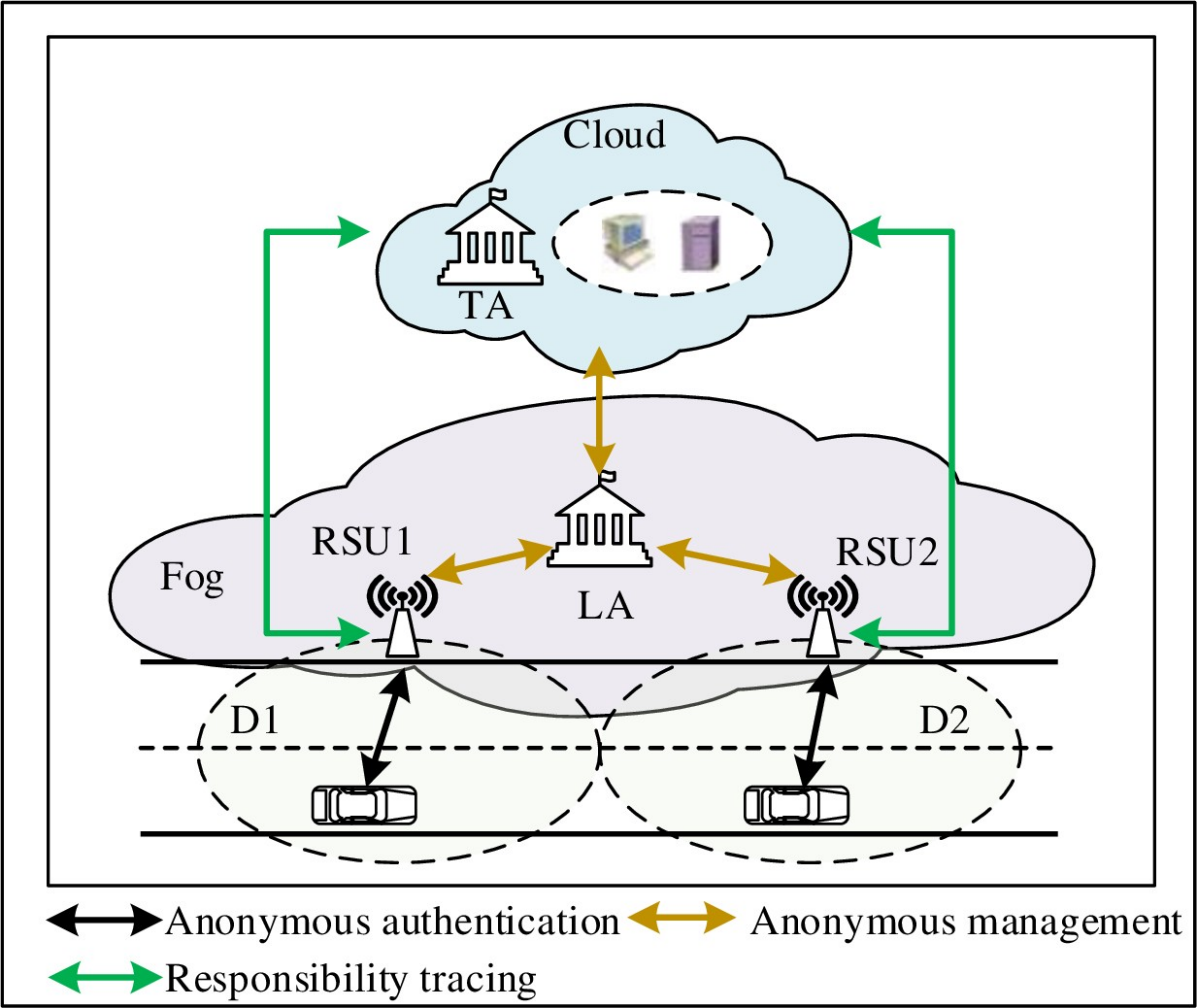
System design.

As depicted in Fig 2, the main design of this system is the anonymous-authentication scheme. It includes the following two processes: system initialization, and efficient and secure authentication scheme. The summary of the symbols used in this paper is provided in Table 1.

**Table 1:** Descriptions of symbols.

Symbol	Descriptions
*U*	Entity *U*, such as the vehicle or the RSU
*TID*_*U*_,*FID*_*U*_	True and anonymous identities of entity *U*
(*PK*_*U*_,*SK*_*U*_)	Public and private keys of entity *U*
*Q*_*U*_,*S*_*U*_	Certification parameters of entity *U*
*HMAC*_*K*_(∙)	Hash message authentication code by using key *K*
*E*_*K*_(∙)	Encrypt the message by using key *K*
*D*_*K*_(∙)	Decrypt the cipher text by using key *K*
$\sigma_{{SK}_{U}}$	Signature of entity *U*
*TS*	Time stamp
*L*_*U*_	Location of *U*
*M*_*i*_	Message

### System initialization

#### Cloud layer

TA: It generates the public parameters, namely, $G_{1},G_{2},G,q,P,e,$ and *p*, and initializes the system by using the following steps [22]:

1. TA chooses a random number, $\psi_{TA}\in z_{q}^{*}$, as the private key, *SK*_*TA*_ = *ψ*_*TA*_, and computes the corresponding public key, *PK*_*TA*_ = *ψ*_*TA*_*P*.
2. TA chooses hash functions $H_{1}:\{0,1\}\to G_{1},\;h:\{0,1\}\to z_{q}^{*}$.
3. TA chooses a security symmetric cryptographic, *E*_*K*_(∙), publishes the system parameters, namely, $G_{1},G_{2},G,q,P,e,p,$ *PK*_*TA*_,*H*(∙),*h*(∙) and *E*_*K*_(∙), following which it downloads the system parameters into fog layer and vehicles.

#### Fog layer

LA: The cloud layer distributes the TID, private key, public key, etc. to the LAs as follows:

1. TA chooses a random number, $\varepsilon_{i}\in z_{p}^{*}$, as its private key and computes the corresponding public key, ${PK}_{{LA}_{i}}=\varepsilon_{i}P$.
2. TA computes the certification parameters, $Q_{{LA}_{i}}=H({TID}_{{LA}_{i}})$, $S_{{LA}_{i}}=\psi_{TA}Q_{R_{i}}$, following which it generates the signature, $\sigma_{SK}({PK}_{{LA}_{i}},{TID}_{{LA}_{i}},L_{{LA}_{i}},h(L_{{LA}_{i}}))$.

RSU: The cloud layer distributes the TID, private key, public key, etc. to the RSUs as follows:

1. TA chooses a random number, $\rho_{i}\in z_{p}^{*}$, as its private key and computes the corresponding public key, ${PK}_{R_{i}}=\rho_{i}P$.
2. TA computes the certification parameters, $Q_{R_{i}}=H({TID}_{R_{i}})$, $S_{R_{i}}=\psi_{TA}Q_{R_{i}}$, and then generates the signature, $\sigma_{SK}({PK}_{R_{i}},{TID}_{R_{i}},L_{R_{i}},h(L_{R_{i}}))$.

#### Vehicles

**Fig 3:**
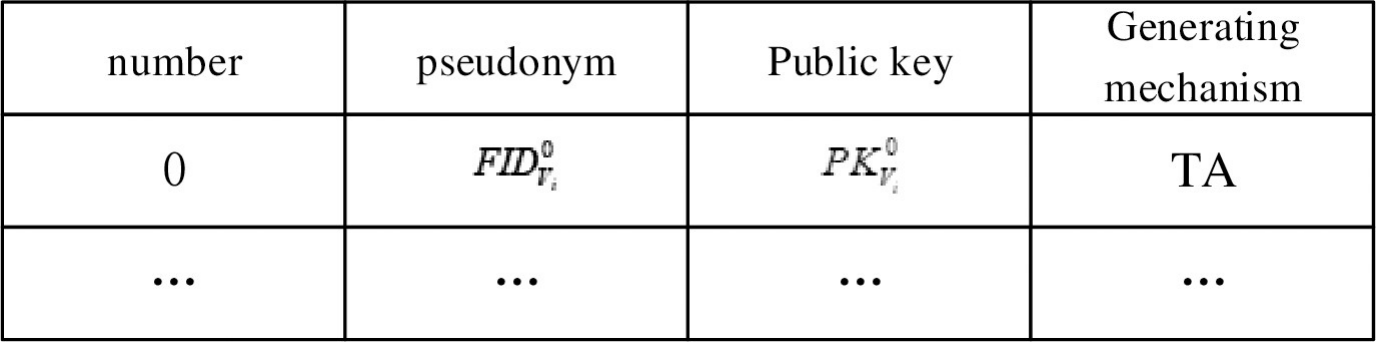
Anonymous tracking table.

TA distributes the pseudonym, private key, public key, etc. to the vehicles as follows:

1. TA computes the certification parameters, $Q_{V_{i}}=H_{1}({TID}_{V_{i}})$ and $S_{V_{i}}=\psi_{TA}Q_{V_{i}}$.
2. TA chooses a random number, $r_{i}\in z_{p}^{*}$, as vehicles’ private key, ${SK}_{V_{i}}=r_{i}$, then generates the public key, ${PK}_{V_{i}}^{0}=r_{i}P$, and pseudonym, ${FID}_{V_{i}}^{0}={TID}_{V_{i}}⨁H_{1}(r_{i}*{PK}_{TA})$, and finally generates certificates, ${\sigma_{{SK}_{TA}}(∙)=\sigma }_{{SK}_{TA}}({FID}_{V_{i}}^{0},{PK}_{V_{i}}^{0})$.
3. TA sends the anonymity of vehicle *V*_*i*_, ${FID}_{V_{i}}^{0}$, and the corresponding public key, ${PK}_{V_{i}}^{0}$, to the storage resource, then generates an anonymous tracking table starting with $\left\{{FID}_{V_{i}}^{0},{PK}_{V_{i}}^{0},TA\right\}$. The anonymous tracking table is depicted in Fig 3.

### Efficient and secure authentication scheme

This study proposes an anonymous-authentication scheme, which is based on pseudonym and fog computing, to meet the efficiency and security requirements in the VANET. First, we design a self-checking authentication, instead of the traditional authentication with reliable authority, thereby improving the efficiency of illegal vehicle authentication. Furthermore, fog computing is introduced to realize anonymous management, reduce the number of authentications, and improve the efficiency of authentication.

**Fig 4:**
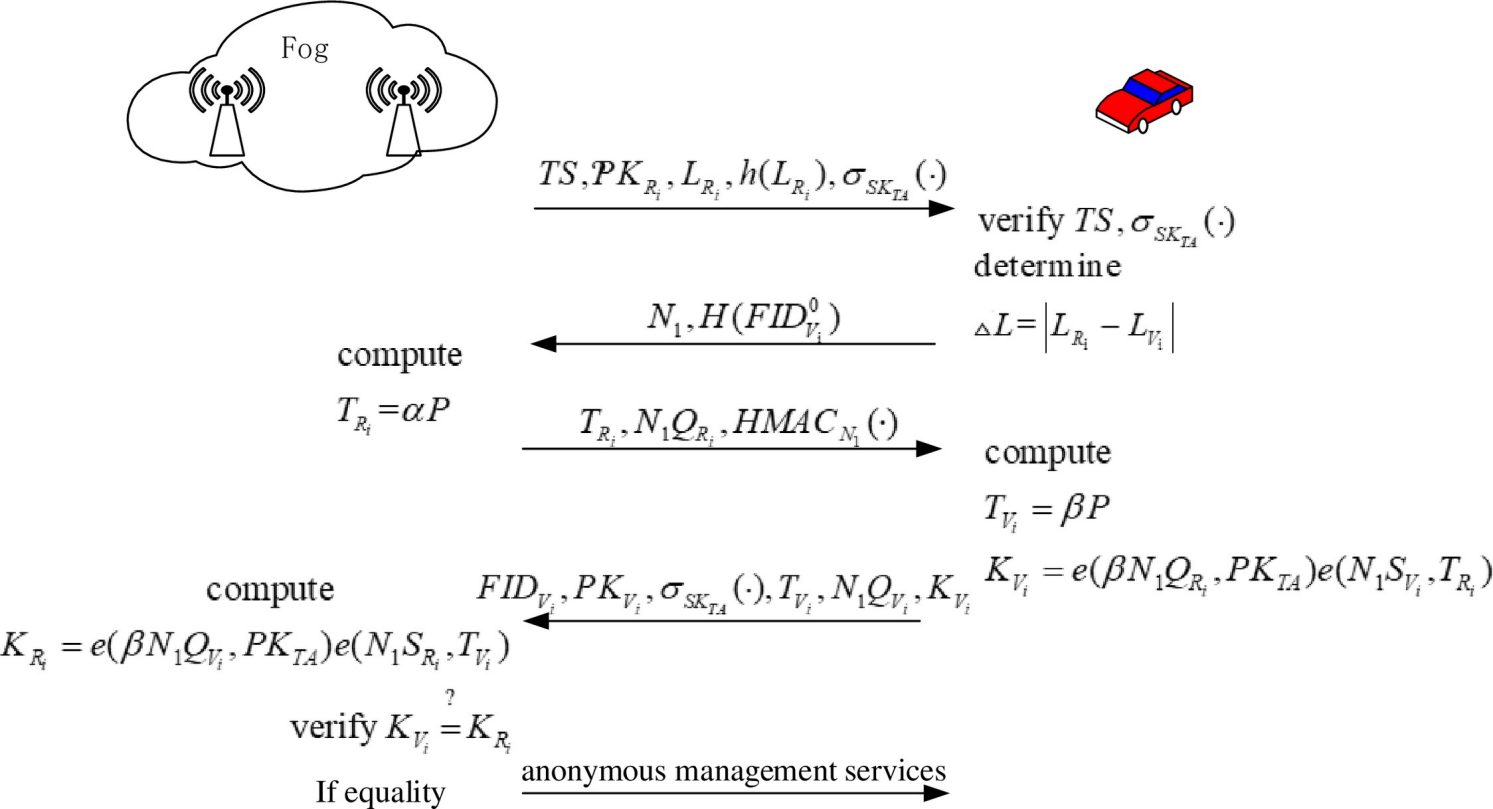
Authentication process for Situation 1.

**Fig 5:**
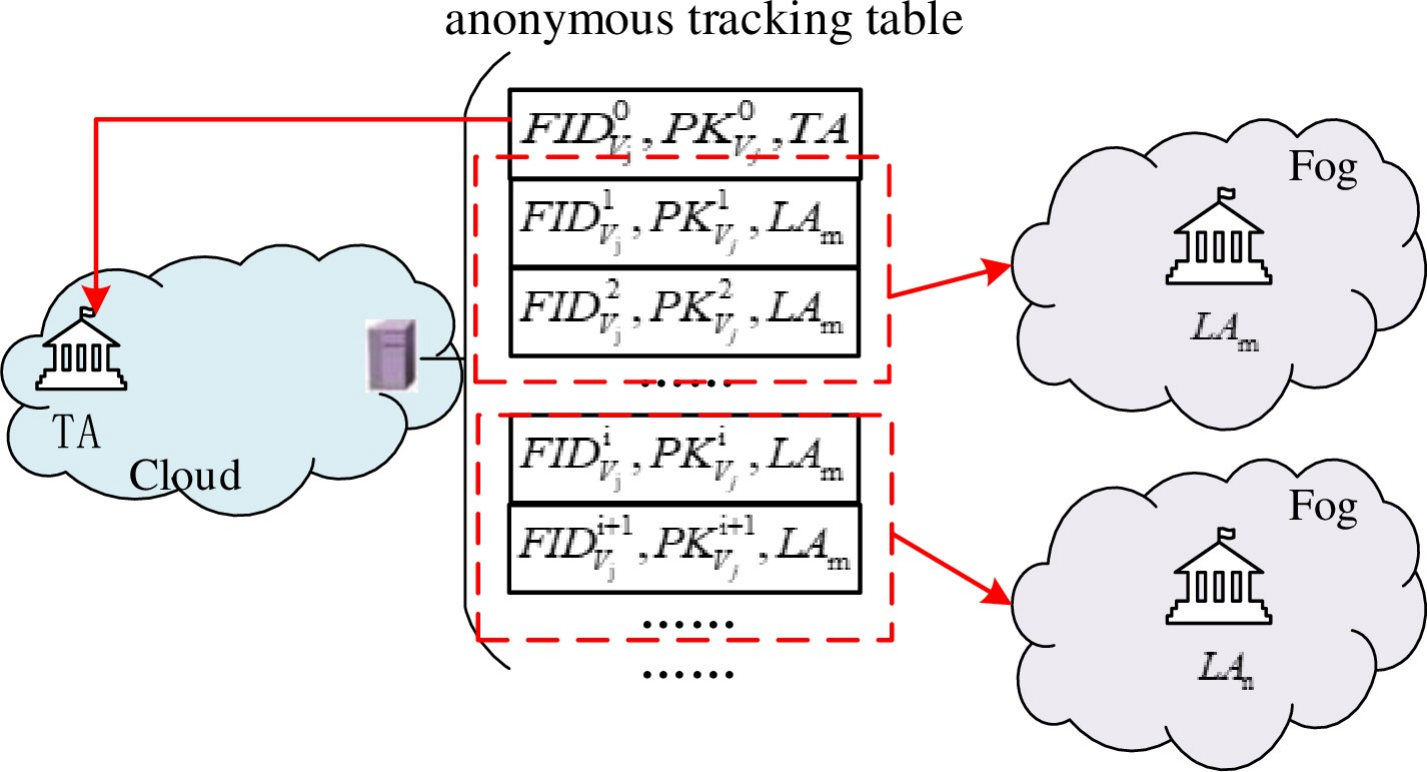
Corresponding pseudonym tracking table in cloud layer.

**Fig 6:**
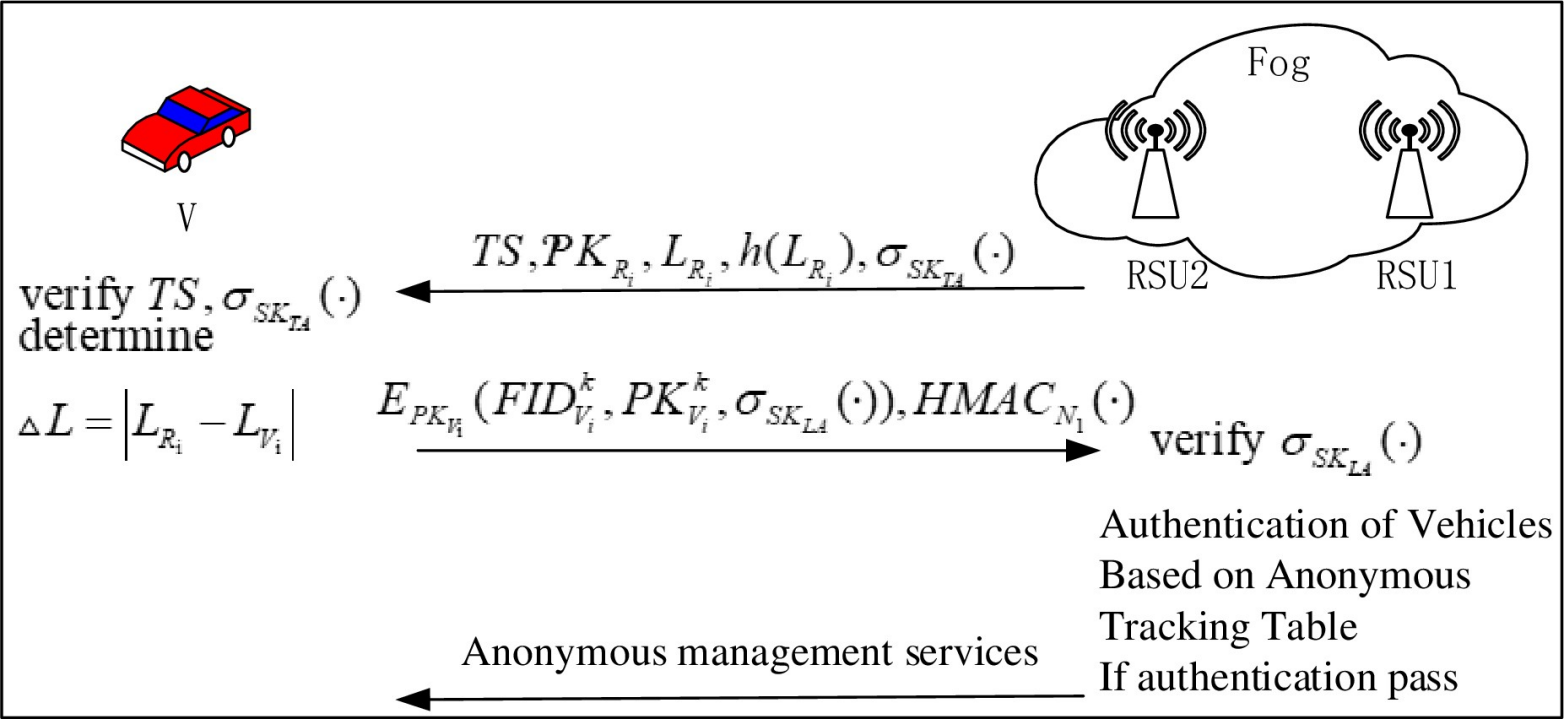
Authentication process for Situation 2.

In this scheme, the authentication process between the fog layer and the vehicle does not require the participation of the cloud layer. In addition, the vehicles are divided according to the following two categories: 1) Situation 1: the previous vehicles have not been certified by other RSUs in the fog layer; and 2) Situation 2: the previous vehicles have been certified by other RSUs in the fog layer. For the vehicles in Situation 1, it can be authenticated by anonymity and authentication parameters, including five information exchanges. However, in Situation 2, the vehicle can be quickly validated by checking the anonymous tracking table, requiring only two rounds of information alternation. Simultaneously, both the authentication processes can achieve anonymous authentication. The information-exchange model and both the main authentication process are depicted in Figs 4, 5 and 6, respectively. In addition, the detailed authentication process is as follows:

RSUs broadcast the messages: RSUs broadcast messages periodically as follows:

$$M_{1}:\;(TS,{PK}_{R_{i}},L_{R_{i}},h(L_{R_{i}}),\sigma_{{SK}_{TA}}({PK}_{R_{i}},L_{R_{i}},h(L_{R_{i}}))).$$

#### Vehicles authenticate the RSUs

When vehicle *V*_*i*_ drives into the domain of *RSU*_*i*_, the former could receive *M*_1_ and verify it as follows:

- *V*_*i*_ receives *M*_1_ and verifies the timestamp *TS* first by computing |*CT*−*TS*|<*Δt*, (*Δt* is the expected network-transmission delay).
- *V_i_* obtains ${PK}_{R_{i}},\;L_{R_{i}}$, and $h(L_{R_{i}})$ from *M*_1_, and, thereafter, it verifies $\sigma_{{SK}_{TA}}({PK}_{R_{i}},L_{R_{i}},h(L_{R_{i}}))$ by using *PK*_*TA*_.
- *V_i_* obtains the current geographic location, $L_{V_{i}}$, from the GPS in vehicles and, subsequently, computes $\Delta L=|L_{R_{i}}-L_{V_{i}}|$ and determines Δ*L* ≤ 600.

Upon completing the entire process, *V*_*i*_ completes the authentication for *RSU*_*i*_.

#### RSUs authenticates the vehicles

Situation 1: The vehicle had not been authenticated by other RSUs previously. (see Fig 4)

- *V*_*i*_ selects a random number, *N*_1_, and, thereafter, sends the message, $M_{2}:\;\left(TS,E_{{PK}_{R_{i}}}(N_{1},H({FID}_{V_{i}}^{0})),{HMAC}_{N_{1}}(∙)\right)$, to *RSU*_*i*_ of the fog layer.
- *RSU*_*i*_ obtains *N*_1_ from *M*_2_ and verifies ${HMAC}_{N_{1}}(∙)$ first; subsequently, it selects a random number, $\alpha_{i}\in z_{p}^{*}$, computes $T_{R_{i}}=\alpha P$ and finally sends the message, $M_{3}:\;(TS,E_{N_{1}}(T_{R_{i}},N_{1}Q_{R_{i}},{HMAC}_{N_{1}}(∙))$, to *V*_*i*_.
- *V*_*i*_ receives *M*_3_ and verifies ${HMAC}_{N_{1}}(∙)$; subsequently, it selects a random number, $\beta_{i}\in z_{p}^{*}$, to compute $T_{V_{i}}=\beta P$ and $K_{V_{i}}=e(\beta N_{1}Q_{R_{i}},{PK}_{TA})e(N_{1}S_{V_{i}},T_{R_{i}})$, following which *V*_*i*_ sends the message, $M_{4}:\;(TS,E_{N_{1}}({FID}_{V_{i}}^{0},{PK}_{V_{i}}^{0},\sigma_{{SK}_{TA}}(),T_{V_{i}},N_{1}Q_{V_{i}},K_{V_{i}},{HMAC}_{N_{1}}(∙)),$ to *RSU*_*i*_.
- *RSU*_*i*_ obtains ${FID}_{V_{i}}^{0},\;T_{V_{i}}$, ${PK}_{V_{i}}^{0},\sigma_{{SK}_{TA}}(∙),$ $N_{1}Q_{V_{i}}$, and $K_{V_{i}}$ from *M*_4_ and, subsequently, verifies ${HMAC}_{N_{1}}(∙)$, following which it calculates the parameters, $K_{R_{i}}=e(\alpha N_{1}Q_{V_{i}},{PK}_{TA})e(N_{1}S_{R_{i}},T_{V_{i}})$. If formula (1) holds, *RSU*_*i*_ completes the authentication for *V*_*i*_. Meanwhile, the fog layer begins to provide anonymous management services. Thus,

$$K_{V_{i}}{=K}_{R_{i}}$$ (1)

When *RSU*_*i*_ completes the authentication of vehicle *V*_*i*_, *RSU*_*i*_ sends the pseudonym of vehicle authentication, as well as the corresponding public key and certificate {${FID}_{V_{i}}^{0},{PK}_{V_{i}}^{0}$} to the local authentication, *LA*_*m*_, in the fog layer.

- *LA*_*m*_ generates *w* numbers of anonymities ${\{FID}_{V_{i}}^{k},{SK}_{V_{i}}^{k},\sigma_{{SK}_{LA}}({FID}_{V_{i}}^{k},{PK}_{V_{i}}^{k}){\}}_{k=1}^{w}$ (where ${SK}_{V_{i}}^{k},\sigma_{{SK}_{LA}}({FID}_{V_{i}}^{k},{PK}_{V_{i}}^{k})$ are pseudonyms corresponding to the private key and pseudonym certificate) for vehicle *V*_*i*_ and, subsequently, sends them to vehicle *V*_*i*_ through *RSU*_*i*_. Simultaneously, *LA*_*m*_ uploads ${\{FID}_{V_{i}}^{k},{PK}_{V_{i}}^{k},\sigma_{{SK}_{LA}}({FID}_{V_{i}}^{k},{PK}_{V_{i}}^{k}){\}}_{k=1}^{w}$ (see Table 2) to TA in the cloud layer.
- TA updates and stores the corresponding pseudonym tracking table of vehicles in the storage resource(the anonymous tracking table is depicted in Fig 5.), and, thereafter, sends ${\{FID}_{V_{i}}^{k},{PK}_{V_{i}}^{k},\sigma_{{SK}_{LA}}({FID}_{V_{i}}^{k},{PK}_{V_{i}}^{k}){\}}_{k=1}^{w}$ to the fog layer. All the RSUs in the fog layer share the updated anonymous table of vehicle *V*_*i*_ through fog calculation in order to reduce the authentication process of other RSUs except that of *RSU*_*i*_.
- RSU updates the new anonymous table of vehicle *V*_*i*_ and deletes the previous anonymous table.

**Table 2:** Anonymities of vehicle *V*_*i*_ generated by *LA*_*m*_.

Pseudonym	Public key	Generating mechanism
${FID}_{V_{i}}^{1}$	${PK}_{V_{i}}^{1}$	*LA*_*m*_
${FID}_{V_{i}}^{2}$	${PK}_{V_{i}}^{2}$	*LA*_*m*_
……	……	……
${FID}_{V_{i}}^{k}$	${PK}_{V_{i}}^{k}$	*LA*_*m*_
……	……	……

Situation 2: The vehicle had previously been authenticated by other RSUs. (see Fig 6)

- *V*_*i*_ sends the message, $M_{2}:\;(TS,\;E_{{PK}_{R_{i}}}({FID}_{V_{i}}^{k},{PK}_{V_{i}}^{k},\sigma_{{SK}_{LA}}({FID}_{V_{i}}^{k},{PK}_{V_{i}}^{k})),{HMAC}_{N_{1}}(∙)$), to *RSU*_*i*_.
- *RSU_i_* receives *M*_2_, first verifies *TS* and *HMAC*_*N*;1_(∙), and then verifies $\sigma_{{SK}_{LA}}({FID}_{V_{i}}^{k},{PK}_{V_{i}}^{k})$. If the verification is successful, the anonymous vehicle is validated according to the pseudonym tracking table sent by the TA.

### Identity tracking

**Fig 7:**
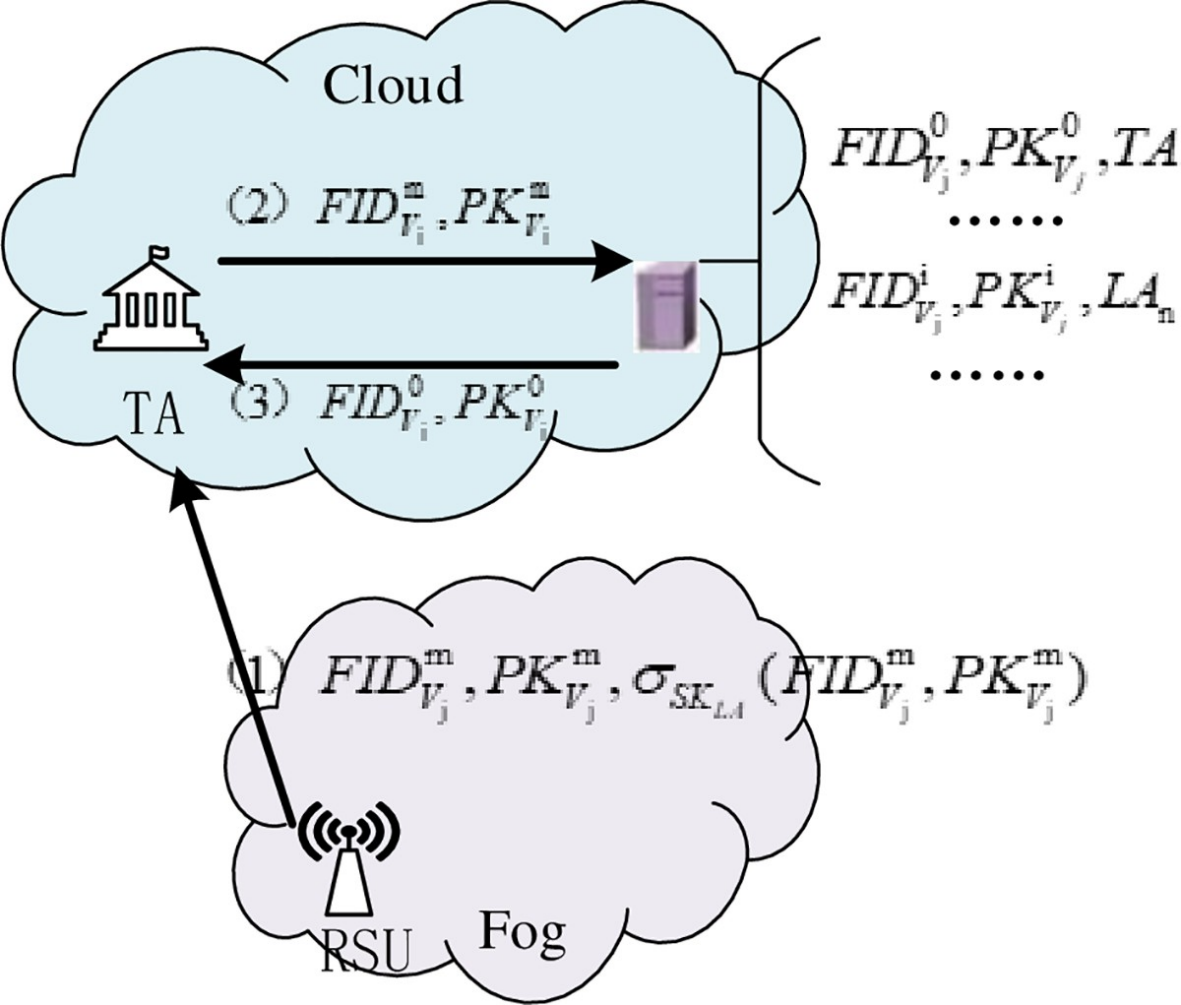
Identity-tracking process.

1. RSU sends the anonymous information, {${FID}_{V_{j}}^{m},{PK}_{V_{j}}^{m},\sigma_{{SK}_{LA}}({FID}_{V_{j}}^{m},{PK}_{V_{j}}^{m})$} (see Fig 7), to TA in the cloud layer after discovering illegal vehicles.
2. TA finds the initial anonymity and other parameters {${FID}_{V_{i}}^{0},{PK}_{V_{i}}^{0}$} of the illegal vehicle according to the anonymous tracking table in the storage resources.
3. Finally, TA tracks the TID of the vehicle, *TID_Vi_*, by computing ${TID}_{V_{i}}={FID}_{V_{i}}^{0}⨁H_{1}({SK}_{TA}*{PK}_{V_{i}}^{0})$.

## Security analysis

In this section, we will provide the security analysis of this study.

### Authentication

The authentication of the proposed scheme is proved using the following two aspects.

#### Authentication of RSU

When a vehicle drive into the domain of an RSU, the former must first authenticate the identity of the latter. In this study, the authentication of the RSU is achieved by the signature and geographic location.

According to the message *M*_1_ sent by the RSU, vehicles first verify signature $\sigma_{{SK}_{TA}}$, following which the vehicles compute $\Delta L=|L_{R_{i}}-L_{V_{i}}|$, and finally determine whether Δ*L*≤600 to ensure the legitimacy of the RSU.

In this process, the signature $\sigma_{{SK}_{TA}}$ is generated by the TA, and the private key of the TA, *SK*_*TA*_, is known only to the TA without any transmission. Therefore, any attacker cannot obtain *SK*_*TA*_ and forge the signature. Thus, only the legitimate RSU has the signature, $\sigma_{{SK}_{TA}}$.

In addition, the value of Δ*L* is calculated using the geographic location of the RSU and vehicles. If the signature, $\sigma_{{SK}_{TA}},$ is correct, the geographic location of the RSU, $L_{R_{i}},$in *M*_1_ is also correct. Meanwhile, the geographic location of vehicles, $L_{V_{i}},$ is obtained from the GPS in the vehicle. Therefore, Δ*L* must be not be more than 600 m (the communication range of the RSU is approximately 600 m).

Consequently, when $\sigma_{{SK}_{TA}}$ is correct and Δ*L*≤600, the identity of the RSU is legal.

#### Authentication of vehicles

Situation 1: The vehicle had previously been authenticated by other RSU.

If the adversary wants to impersonate a legal vehicle to get authenticated by the RSUs, it must generate a valid message, *M*_4_, and send it to the RSUs. According to *M*_4_, the RSUs will verify the legality of the vehicular identity on the basis of formula (2) and *K*_*V*_ in message, *M*_4_. One has

$$\begin{array}{l} K_{V}=e\left(\beta N_{1}Q_{R},PK_{TA}\right)e\left(N_{1}S_{V},T_{R}\right)\\ \;=e\left(\beta N_{1}H_{1}\left(TID_{R}\right),\psi_{TA}P\right)e\left(N_{1}\psi_{TA}H_{1}\left(TID_{V}\right),\alpha P\right)\\ \;=e\left(\alpha N_{1}H_{1}\left(TID_{V}\right)+\beta N_{1}H_{1}\left(TID_{R}\right),\psi_{TA}P\right)\\ \;=e\left(\alpha N_{1}Q_{V_{i}},PK_{TA}\right)e\left(N_{1}S_{R_{i}},T_{V_{i}}\right)\\ \;=K_{R_{i}} \end{array}$$ (2)

If formula (2) is workable, the identity of the vehicles is legal.

Situation 2: The vehicle had previously been authenticated by other RSUs.

If the vehicle had previously been authenticated by RSU_*i*−1_, then RSU_*i*_ only needs to authenticate it according to the anonymous tracking table sent by the cloud.

Theorem: Assuming that H is a random oracle, the DL and CDH assumptions are valid, and the identities of the vehicles in this scheme are authenticated.

Proof: If an adversary, A, could impersonate a real identity of a legal vehicle, *TID*_*V*_, and generate a valid message, *M*_4_, then it must be able to compute the valid value of the parameter *S*_*V*_ = *sP* = *ψ*_*TA*_*Q*_*V*_*P* = *ψ*_*TA*_*H*_1_(*TID*_*V*_)*P*. The advantage of the success of A is ${Adv}_{A}^{M4}$.

Constructing two algorithms, $D_{CDH}$ and $D_{DL}$, to solve the CDH and DL problems, respectively.

**Game 1**:

Setup: According to the section system initialization, $D_{DL}$ generates the public parameters, namely, $G_{1},G_{2},P,q,e,G,p,g,\;{PK}_{TA},H_{1}(∙),h(∙),\;and\;E_{k}(∙)$, and sends them to A. Subsequently, A could query $D_{DL}$ up to *q*_*DL*_ times.

**Query**:

1. A queries what is *TID*_*V*_ equal to?
2. $D_{DL}$ defines *s* = *H*_1_(*TID*_*V*_)*P* and returns it to A.

**Challenge 1**:

1. After A received *s*, it inputs (*P*,*s*) to obtain *H*_1_(*TID*_*V*_) by $D_{DL}$;
2. A inputs (*P*,*PK*_*TA*_) to obtain *ψ*_*TA*_;
3. A computes *S*_*V*_ = *ψ*_*TA*_*H*_1_(*TID*_*V*_)*P*.

In the above-mentioned process, the advantage of the success of A is ${Adv}_{A}^{DL}=2q_{DL}∙{Adv}_{DL}$.

**Game 2**:

Setup: According to the section system initialization, $D_{DL}$ generates the public parameters, $G_{1},G_{2},P,q,e,G,p,g,\;{PK}_{TA},H_{1}(∙),h(∙),\;{and\;E}_{k}(∙),$ and sends them to A. Thereafter, A could query $D_{CDH}$ up to *q*_*CDH*_ times.

**Query**:

1. A queries what is *TID*_*V*_ equal to?
2. $D_{CDH}$ defines *s* = *H*_1_(*TID*_*V*_)*P* and returns it to A.

**Challenge 2**:

After A receives *s*. it inputs (*P*, *PK*_*TA*_, *s*) to obtain *H*_1_(*TID*_*V*_) by $D_{CDH}$.

In the above-mentioned process, the advantage of the success of A is ${Adv}_{A}^{CDH}=q_{CDH}∙{Adv}_{CDH}$.

In summary, the advantage of A generating the valid message, *M*_4_, i.e., the advantage of successfully calculating the valid parameter *S*_*V*_ is as follows:

$$\begin{array}{l} Adv_{A}^{M_{4}}=Adv_{A}^{DL}+Adv_{A}^{CDH}\\ \;=2q_{DL}\cdot Adv_{DL}+q_{CDH}\cdot Adv_{CDH} \end{array}$$

According to the section definitions and assumptions, the advantage of $D_{DL}$ successfully solving the DL problem and that of $D_{CDH}$ successfully solving the CDH problem, in polynomial time, can be neglected. Thus, the advantages of A successfully generating a valid message, *M*_4_, is also negligible.

Therefore, the identity of the vehicle in Situation 1 satisfies the authentication requirement of the node identity. However, if the vehicle had previously been authenticated by *RSU*_*i*±1_ (Situation 2), then the latter only needs to authenticate the former according to the anonymous tracking table sent by the cloud.

### Anonymity of vehicles

The anonymity of the proposed scheme is realized by the anonymous management of cloud and fog.

Sensitive information such as ${FID}_{V_{i}}$ is included in the information sent by vehicles. In clouds, because the private key of the TA is secure, except for that of vehicles, only the TA knows the real identity of the vehicles; therefore, attackers cannot forge pseudonyms issued by clouds. In addition, only the TA in the cloud can reveal the relationship between vehicle anonymity and real identity, when illegal vehicles are found. In the fog layer, the RSU can authenticate the vehicle anonymously without knowing the real identity of the vehicle. Simultaneously, because the LA generates a pseudonym without obtaining the real name of the legitimate vehicle and uploads the pseudonym to the cloud, it cannot be traced back to obtain the real name of the vehicle. Therefore, no attacker can obtain the real identity of the vehicle.

### Traceability

Vehicles communicate with the RSU by using their anonymities, and some malicious vehicles may send false information to cause traffic accidents. In this situation, the cloud layer can reveal the identity of the vehicles with the help of the TA and the storage resources of the cloud layer.

After receiving the anonymous, {${FID}_{V_{j}}^{m},{PK}_{V_{j}}^{m},\sigma_{{SK}_{LA}}(∙)$}, of the irregular vehicle V sent by the RSU, the cloud finds the initial anonymous, {${FID}_{V_{i}}^{0},{PK}_{V_{i}}^{0}$}, of vehicle V in the anonymous tracking table of the storage resources. Thereafter, the initial anonymity is sent to the TA. When the TA receives {${FID}_{V_{i}}^{0},{PK}_{V_{i}}^{0}$}, it obtains the real name, *TID*_*V*_, of the illegal vehicle according to the following formula:

$$\begin{array}{l} TID_{V}=FID_{V_{i}}^{0}⊕H_{1}\left(SK_{TA}*PK_{V_{i}}^{0}\right)\\ \;=TID_{V_{i}}⊕H_{1}(r_{i}*PK_{TA})⊕H_{1}\left(SK_{TA}*PK_{V_{i}}^{0}\right)\\ \;=TID_{V_{i}} \end{array}$$ (3)

Thus, the traceability of the proposed scheme is achieved.

### Message integrity and unforgeability

In the VANET, messages are more likely to become invalid requests, such as packet loss or bogus messages forged by attackers, as the communication model between the vehicles and RSU or among the vehicles, is based on wireless communication. To ensure the integrity of the messages, most existing schemes utilize HMAC or signature. In this study, the integrity of the messages can be achieved using HMAC because of its lightweight overhead.

In this study, the unforgeability of the messages is achieved by $\sigma_{{SK}_{TA}}$ or ${HMAC}_{N_{1}}(∙)$. In message *M*_1_, the signature, $\sigma_{{SK}_{TA}},$ is generated by the TA by using its private key *SK*_*TA*_. Because *SK*_*TA*_ is only held by the TA, attackers cannot compute it according to the public key *PK*_*TA*_ = *ψ*_*TA*_*P*. Thus, $\sigma_{{SK}_{TA}}$ cannot be unforged by attackers. In messages *M*_2_−*M*_5_, *N*_1_ is the shared key between the vehicles and RSU. Vehicles encode it using the public key of the RSU and then send it back. However, only the RSU can decode it using its private key, *SK*_*RSU*_, and obtain *N*_1_. Thus, attackers are unable to gain *N*_1_ and forge messages.

## Performance evaluation

In this section, we evaluate the performance of the proposed scheme. First, we compare the proposed scheme with the existing schemes in terms of computation and communication costs. In addition, we evaluated the average delay of the proposed scheme.

### Computation cost analysis and comparison

According to reference [23], the computation cost mainly depends on the following three parameters: first, the time taken to execute a pairing operation, *T*_*p*_; second, the time taken to execute one-point multiplication over an elliptic curve, *T*_*m*_; and third, the time taken to execute a MapToPoint hash function, *T*_*h*_, where *T*_*p*_ = 1.6 ms, *T*_*m*_ = 0.6 *ms* and *T*_*h*_ = 2.7*ms*. This paper does not consider other operations requiring low computational costs, such as the HMAC operation (executing time is 0.006 ms).

**Fig 8:**
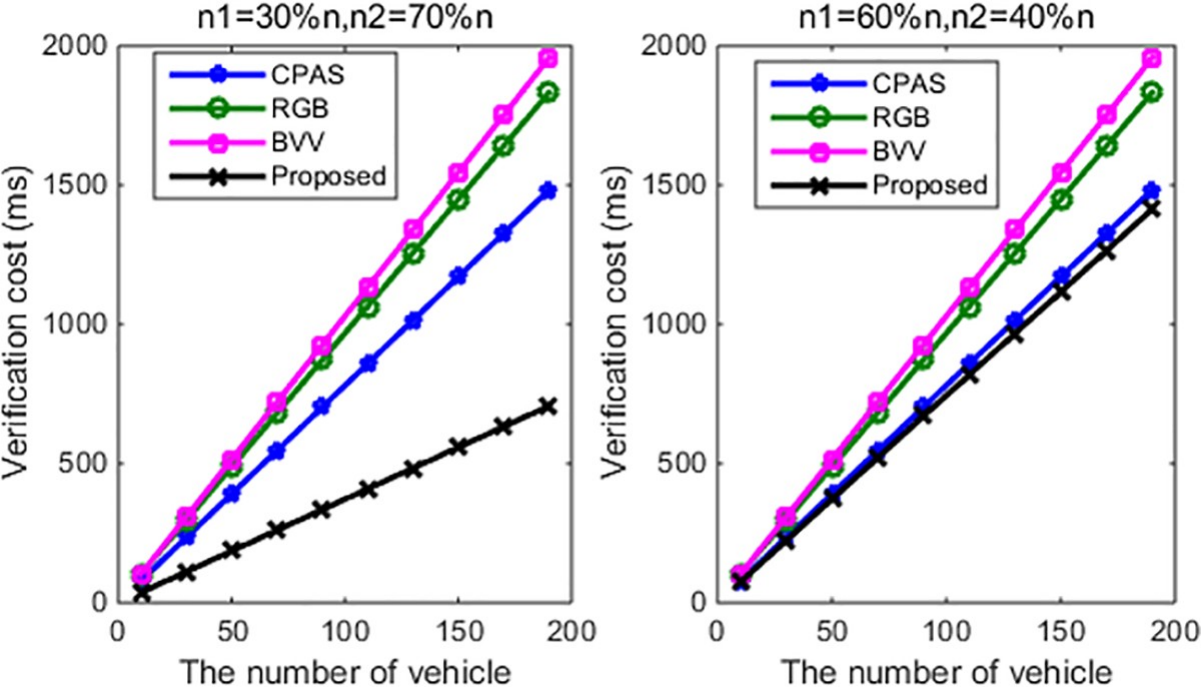
Number of vehicles and verification cost.

Because this study divides vehicles into two categories, both of which have has been mentioned previously, the number of vehicles needed to be verified, *n*, includes the number of vehicles in Situation 1, *n*_1_, and that in Situation 2, *n*_2_; therefore, *n* = *n*_1_+*n*_2_. Furthermore, Table 3 and Fig 8 objectively illustrate the comparison between our proposed scheme and other existing schemes, in terms of the verification time. From Fig 8, it can be observed that our proposed scheme requires lower computational cost. Especially, when the number of vehicles, *n*, is equal to 100, the CPAS, RGB, and BVV take 780.6, 968.1, and 1030 ms, respectively. Whereas the proposed scheme takes only 448 ms (*n*_1_ = 30%*n*, *n*_2_ = 70%*n*), or 744 ms (*n*_1_ = 60%*n*,*n*_2_ = 40%*n*).

**Table 3:** Verification cost of various schemes.

Scheme	Verify a vehicle	Verify *n* vehicles (*n* = *n*_1_+*n*_2_)
CPAS	5*T*_*m*_+3*T*_*p*_	(5*n*+1)*T*_*m*_+3*nT*_*p*_
RGB	3*T*_*m*_+*T*_*h*_	(7*n*+1)*T*_*m*_+(2*n*+1)*T*_*h*_
BVV	3*T*_*h*_+*T*_*m*_+*T*_*p*_	3*nT*_*h*_+*nT*_*m*_+*nT*_*p*_
Proposed	10*T*_*m*_+4*T*_*p*_ or 0	*n*_1_(10*T*_*m*_+4*T*_*p*_)+*n*_2_0

### Communication-cost analysis and comparison

In this paper, the communication cost is represented by the size of messages. In this section, we mainly focus on the additional communication cost, such as the cost associated with signature, certification, and pseudonym. As shown in Table 4, the additional sizes of messages are 101 bytes for CPAS [14], 63 bytes for RGB [15], 280 bytes for BVV [17], and 76 bytes for our proposed scheme.

**Table 4:** Comparison of communication cost.

Scheme	Send n message (bytes)
CPAS	101n
RGB	63n
BVV	280n
Proposed	76n

**Fig 9:**
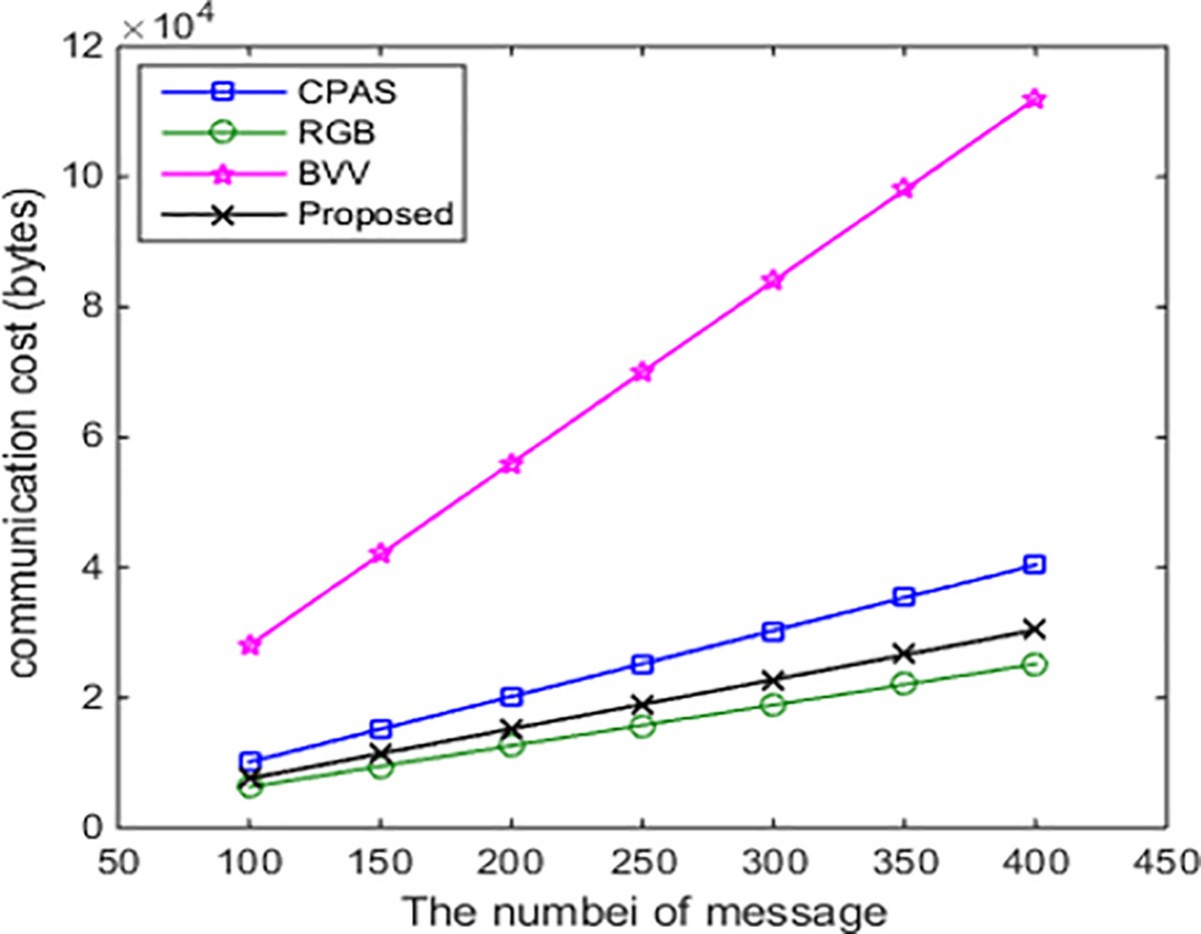
Number of vehicles and communication cost.

In addition, Fig 9 compares the communicational cost of the proposed scheme with those of some existing schemes. From the figure, we can see that the communicational cost of the proposed scheme is lower than that of each CPAS and BVV.

### Experiment and simulation

To ensure the authenticity and feasibility of the experiment, the relevant parameters of this experiment are based on the real data provided by the federal government of the United States [24]. All the simulation parameters are listed in Table 5.

**Table 5:** Simulation parameters.

Parameter	value
Simulation tool	NS2.34
Wireless protocol	802.11p
Channel bandwidth	6 Mb/s
Simulation time	30 s
Road length	1000 m
Communication range of RSU	600 m
Message size	200 bytes
Speed of vehicle	0–30 m/s

#### Average delay

**Fig 10:**
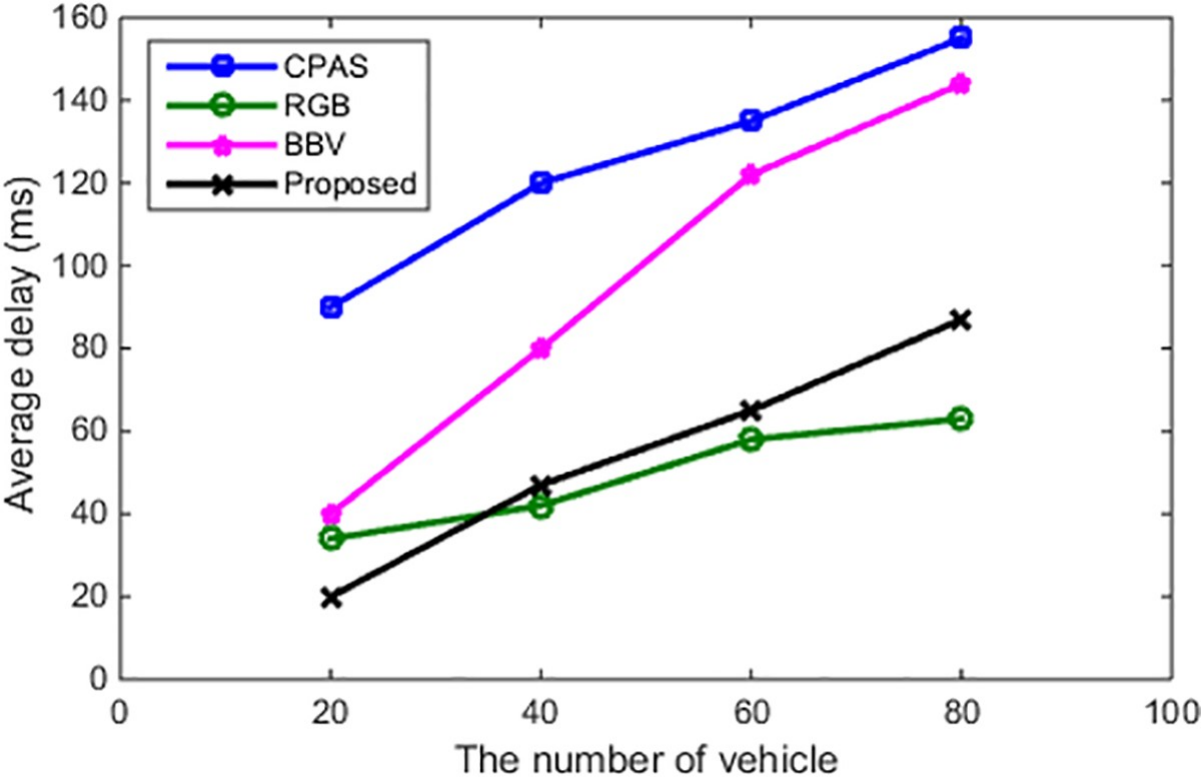
Number of vehicles and average delay.

This study uses formula (4) [25] for evaluating the average delay, where n denotes the number of vehicles, *M*_*i*_ the number of messages sent by the vehicles, $T_{creat}^{n\_m}$ the time in which a vehicle or RSU creates the message m, $T_{communication}^{n\_m\_k}$ the communication time in which the entity (vehicle or RSU) *N* sends the message to the entity *k*, and $T_{verify}^{n\_m\_k}$ the time in which the entity *k* verifies the message *m* from the entity *n*. The average delays for different number of vehicles are depicted in Fig 10. One has

$${Delay}_{ave}=\frac{1}{n}\sum_{i=1}^{n}\frac{1}{M_{i}}\sum_{m=1}^{M}\left(T_{creat}^{N_{m}}+T_{communication}^{N_{m_{k}}}+T_{verify}^{N\_m\_k}\right)$$ (4)

From Fig 10, it can be clearly seen that the average delays of both the RGB and proposed scheme are less than those of the CPAS and BVV, with the same number of vehicles. In addition, our proposed scheme is the most efficient of all the schemes mentioned when the number of vehicles ranges from 20 to 30. Furthermore, when the number of vehicles is more than 30, our proposed scheme becomes more efficient than CPAS and BVV as well.

#### Packet-loss rate

**Fig 11:**
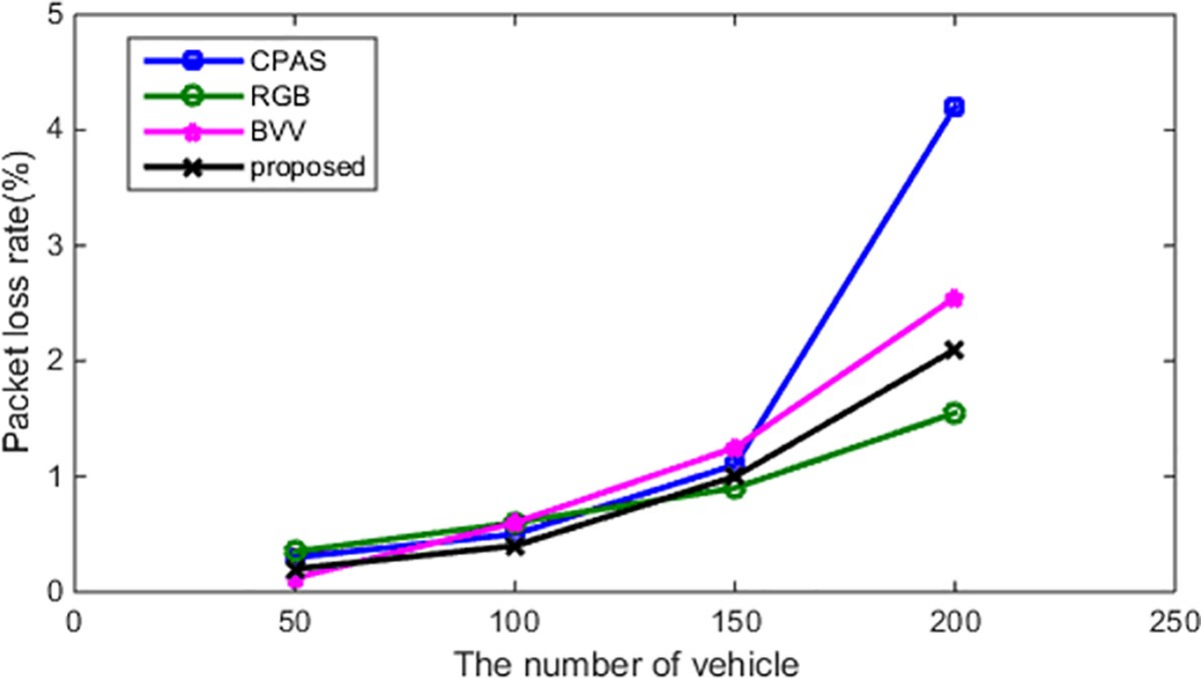
Number of vehicles and the packet-loss rate (static state).

**Fig 12:**
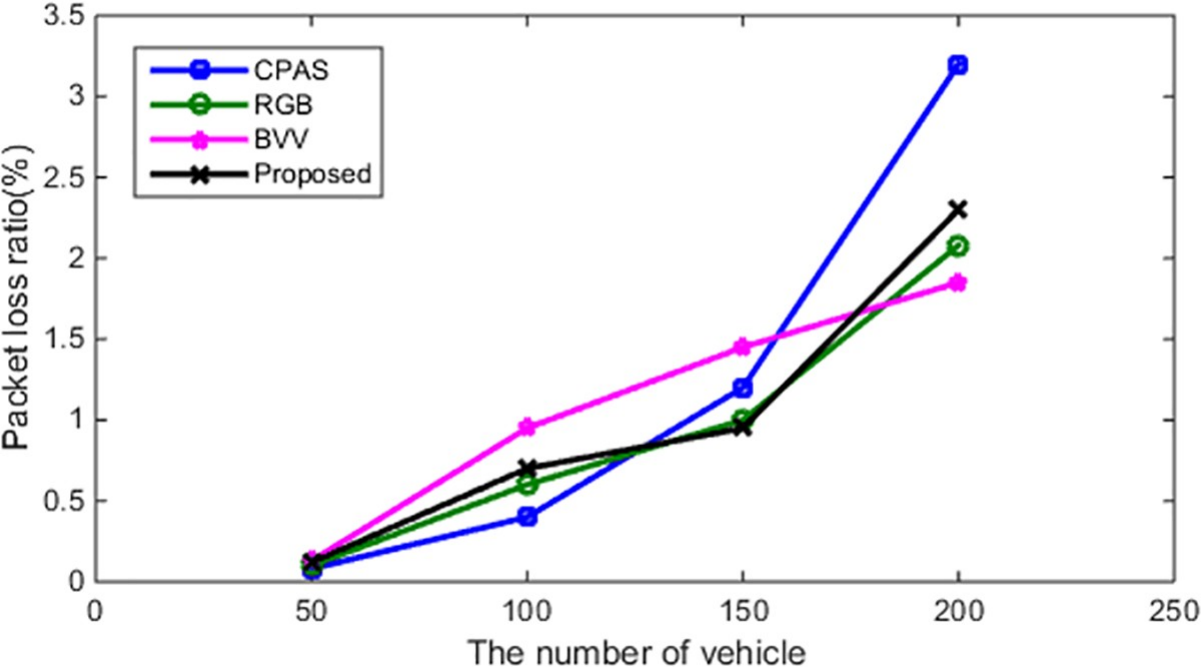
Number of vehicles and the packet-loss rate (dynamic state).

In addition to analyzing the average delay of the proposed scheme, the packet-loss rate of the proposed scheme is compared with that of various schemes, under two states, i.e., static and dynamic states (see Table 6). As depicted in Figs 11 and 12, the proposed scheme also has some advantages with respect to the packet-loss rate.

**Table 6:** Number of vehicles and packet-loss rate.

	Static state (%)	Dynamic state (%)
50	0.2	0.12
100	0.4	0.7
150	1.0	0.95
200	2.1	2.3

## Conclusions

This study presented a fog-computing-based anonymous-authentication scheme for the VANET. In the proposed scheme, vehicles are divided according to two situations. According to the different above-mentioned situations, the RSUs in the fog layer are authenticated using pseudonyms. The pseudonym management of the vehicles is achieved via fog computing, which improves the performance after entering the first authentication, thus realizing both privacy protection and efficient authentication.
